# Outcomes following arthroscopic treatment for femoroacetabular impingement syndrome in patients with ossified labrum

**DOI:** 10.1093/jhps/hnaf033

**Published:** 2025-08-04

**Authors:** Sarantos Nikou, Arvin Nabi, Ida Lindman, Louise Karlsson, Axel Öhlin, Eric Hamrin Senorski, Mikael Sansone

**Affiliations:** Department of Orthopaedics, Institute of Clinical Sciences, Sahlgrenska Academy, University of Gothenburg, Göteborgsvägen 31, 43180, Mölndal, Gothenburg, Sweden; Department of Orthopaedic Surgery, South Älvsborg Hospital, 501 82 Borås, Sweden; Department of Orthopaedics, Institute of Clinical Sciences, Sahlgrenska Academy, University of Gothenburg, Göteborgsvägen 31, 43180, Mölndal, Gothenburg, Sweden; Department of Orthopaedics, Institute of Clinical Sciences, Sahlgrenska Academy, University of Gothenburg, Göteborgsvägen 31, 43180, Mölndal, Gothenburg, Sweden; Research, Education, Development & Innovation, Primary Health Care, Region Västra Götaland, Vänerparken 21, 46280 Vänersborg, Västra Götalands län, Sweden; Department of Orthopaedics, Institute of Clinical Sciences, Sahlgrenska Academy, University of Gothenburg, Göteborgsvägen 31, 43180, Mölndal, Gothenburg, Sweden; Department of Orthopaedics, Institute of Clinical Sciences, Sahlgrenska Academy, University of Gothenburg, Göteborgsvägen 31, 43180, Mölndal, Gothenburg, Sweden; Department of Health and Rehabilitation, Institute of Neuroscience and Physiology, Sahlgrenska Academy, University of Gothenburg, Arvid Wallgrens Backe, Hus 1, 41346, Gothenburg, Sweden; Department of Orthopaedics, Institute of Clinical Sciences, Sahlgrenska Academy, University of Gothenburg, Göteborgsvägen 31, 43180, Mölndal, Gothenburg, Sweden

## Abstract

**Purpose:**

To evaluate the results of arthroscopic treatment for femoroacetabular impingement syndrome (FAIS) with concomitant labrum ossification (LO) using patient-reported outcome measures (PROMs). The hypothesis was that at 2-year follow-up, an improvement in PROMs would be observed.

**Methods:**

A group of 24 patients (28 hips) with FAIS and LO findings were eligible for inclusion. In total 14 patients (18 hips) were included in the final PROMs analysis. The International Hip Outcome Tool short version (iHOT-12) was the primary outcome. The Copenhagen Hip and Groin Outcome Score (HAGOS), the European Quality of Life–5 Dimensions Questionnaire (EQ-5D), the EQ–visual analogue scale (EQ VAS), the Hip Sports Activity Scale for physical activity level, the VAS for overall hip function and a single question regarding overall satisfaction with the surgery were the secondary outcomes.

**Results:**

At 2-year follow-up 6 out of 24 (25%) patients had undergone total hip arthroplasty (THA). Comparing PROMs preoperatively with 2-years follow-up for the group of patients that did not undergo THA (*n* = 14) showed statistically significant improvements (*P* < .05) for: iHOT-12 (39.3 versus 69.1), HAGOS subscales (symptoms 49.0 versus 70.9, pain 53.6 versus 80.0, sport 42.0 versus 68.5, daily activity 53.6 versus 73.2, physical activity 31.7 versus 61.5, quality of life 34.2 versus 65.0), EQ-5D (0.7 versus 0.8) and VAS for overall hip function (44.3 versus 66.5).

**Conclusion:**

Patients with FAIS and LO who had not undergone THA at 2-year follow-up following arthroscopic treatment showed improved outcomes regarding patient-reported outcomes. Level of evidence: Case series, level IV.

## INTRODUCTION

Femoroacetabular impingement syndrome (FAIS) is a common cause of hip and groin pain. There are two types of morphological changes related to femoroacetabular impingement (FAI), either at the head–neck junction (cam) or at the acetabular rim (pincer). While the aetiology of cam is considered to be due to excessive load at the anterosuperior aspect of the proximal femur, the aetiology behind pincer is not fully understood [1, 2]. It is well established that patients with FAI morphology, especially cam, have an increased risk of developing osteoarthritis (OA) [3, 4]. However, little is known on the impact of acetabular based lesions on hip OA [5].

The labrum has an important function providing a sealing effect, thus increasing the cartilage consolidation and hip joint stability, increasing the hip joint contact area and decreasing cartilage stress and strain [6]. Osteophyte formation in joints, including the hip, is common in patients with OA [7]. In some cases, osteophytes occur in and around the hip labrum, which in itself can be partially or completely ossified [8]. This can lead to a local or global pincer morphology, which can cause impingement and related symptoms (Figs 2–4) [7]. The term ‘captured hip’ has been used to describe the state where extensive labrum ossification (LO) leads to restricted range of motion, pain and dysfunction of the hip joint [9]. While osteophytes are a well-known entity, little is known about LO. The distinction between osteophytes due to OA and LO without any other manifestations of OA is still unknown. In LO cases, there is local ossification of the labrum without any remaining labral tissue in this area [10–12]. This distinguishes LO from an osteophyte, where the bony prominence is in most cases covered with labral tissue [13–15].

There is limited evidence on the outcomes after hip arthroscopic treatment for patients with LO. The primary aim of this study is to evaluate the results of arthroscopic treatment for FAIS with concomitant LO using patient-reported outcome measures (PROMs). Our hypothesis was that patients undergoing hip arthroscopy for FAIS in the setting of LO would have improved patient-reported outcomes.

## MATERIALS AND METHODS

A group of 24 patients (28 hips) with FAIS and LO findings that underwent hip arthroscopy between January 2011 and December 2019 were identified in the local hip arthroscopy registry and included in the present study. The registry includes all patients undergoing hip arthroscopy at two hospitals in Gothenburg. The inclusion criteria were patients with FAIS (cam, pincer, or mixed type) and LO. Patients that had undergone total hip arthroplasty (THA) at 2-year follow-up were included initially but excluded from the final PROMs analysis. Patients lost to follow-up were also excluded from the final PROMs analysis. The LO was identified during surgery, and no cases of os.acetabuli or labral calcification were included. The patient inclusion information was retrieved from operative reports. All the data were prospectively collected. All patients had failed to respond to non-operative treatment with physiotherapy before proceeding to surgery. All surgeries included in this study were performed by two high-volume surgeons, performing over 100 hip arthroscopies per year. The patients completed the following PROMs both preoperatively and at the 2-year follow-up: International Hip Outcome Tool short version (iHOT-12), Copenhagen Hip and Groin Outcome Score (HAGOS), European Quality of Life–5 Dimensions Questionnaire (EQ-5D) and EQ–Visual Analogue Scale (EQ VAS), Hip Sports Activity Scale (HSAS), a VAS scale for hip function and a question regarding satisfaction with surgery [16–19]. The demographic and perioperative information, including age, BMI, sex, uni- or bilateral surgery, operated side, surgical and traction time, and Konan classification grade of acetabular chondral damage, were retrieved from patient medical records [20]. The preoperative radiographic parameters of alpha angle, lateral centre-edge angle, Tönnis angle, Tönnis grade of OA, joint space measurement, were reported. Information about reoperations and conversion to THA was also registered. The mean time to conversion to THA was reported.

## SURGICAL TECHNIQUE

The hip arthroscopy procedures were performed in a supine position using one anterolateral and one midanterior portal. To gain access to the central compartment, axial traction was used. Traction is used for central compartment inspection of cartilage procedures, whilst bone resection and labral suture is done mainly without traction. The peripheral and central compartment were approached through a capsulotomy made longitudinally to the capsule fibres and the iliofemoral ligament [21–23]. This approach decreases the potential risk of iatrogenic induced hip laxity postoperatively [24, 25]. The ossified labrum was resected to the perceived anatomical margin of the acetabulum, and in cases with large pincer lesions and remaining labrum tissue, the labrum was reattached with suture anchors (Figs 5 and 6). No labral reconstructions were performed. Postoperatively, full weight-bearing was allowed immediately, but patients were recommended to use crutches for up to 4 weeks when walking outdoors. All patients were assigned to supervised rehabilitation. The patients were furthermore prescribed non-steroid anti-inflammatory drugs 3 weeks postoperatively to prevent heterotopic ossification.

## STATISTICS

Descriptive statistics were used to analyse continuous demographic variables and presented as mean with the standard deviation (SD) and median with range. Nominal data were reported as numbers and percentages. The Statistical Package for the Social Sciences (IBM SPSS statistics, version 28.0.1.1) was used to statistically analyse the patient data and PROMs. Non-parametric statistical testing was used to compare paired means for continuous PROM data not normally distributed. To compare PROMs between preoperative and 2-year follow-up, the Wilcoxon signed rank test was used. The level of significance was set at *P* ˂ .05. Patients exceeding the minimal clinically important difference (MCID) were reported, with the use of a distribution-based technique, setting the cut-off value at 0.5 times the SD of the score change [26]. The number of patients achieving the acceptable symptomatic state (PASS) for the six HAGOS subscales and the iHOT-12 was reported. In previous studies the PASS values are estimated and set for the iHOT-12 63, HAGOS pain 68.8, HAGOS symptoms 62.5, HAGOS function 82.5, HAGOS physical activity 43.8, HAGOS sports 60.9 and HAGOS quality of life 42.5 [18, 27–29].

## RESULTS

There were 24 patients (19 male, 5 female), 28 hips in total, included in the study. The average age at time of surgery was 47.7 years (±8.1 SD) with a mean symptom duration before surgery of 56.4 months (±58.2 SD). Four patients were operated on bilaterally, either on the same occasion (three patients) or on two different occasions (one patient). Demographic and perioperative data are presented in Table 1 and radiographic parameters in Table 2.

**Table 1:** Patient demographics and perioperative data.

Number of patients	24
Number of hips	28
Male/female (%)	79.2/20.8
Age, years mean (SD)	47.7 (8.1)
BMI-mean (SD)	26.3 (1.5)
Unilateral/bilateral surgery (%)	83.3/16.7
Right/Left hip (%)	46.4/53.6
Symptom duration in months, mean (SD)	56.4 (58.2)
Surgery time in minutes, mean (SD)	58.0 (17.5)
Traction time in minutes, mean (SD)	5.1 (6.0)
Konan classification 1a/3a/4a/4b/4c (%)	7.1/28.6/21.4/21.4/21.4^a^
Konan classification 3a/4a/4b/4c (%)	16.7/16.7/33.3/33.4^b^

a The proportion is based on 18 hips included in final PROM analysis.

b The proportion is based on 10 hips excluded from final PROM analysis.

**Table 2:** Preoperative radiographic parameters (24 patients/28 hips).

Alpha angle (degrees)	70 (±9)
CE angle (SD) degrees	32 (±7)
Tönnis angle	6(±3)
Tönnis grade 0 (no hips)	6
Tönnis grade 1 (no hips)	8
Tönnis grade 2 (no hips)	8
Tönnis grade 3 (no hips)	6
Any joint space of 1 (mm)	7
Any joint space of 2 (mm)	15
Any joint space of 3+ (mm)	6

**Figure 1:**
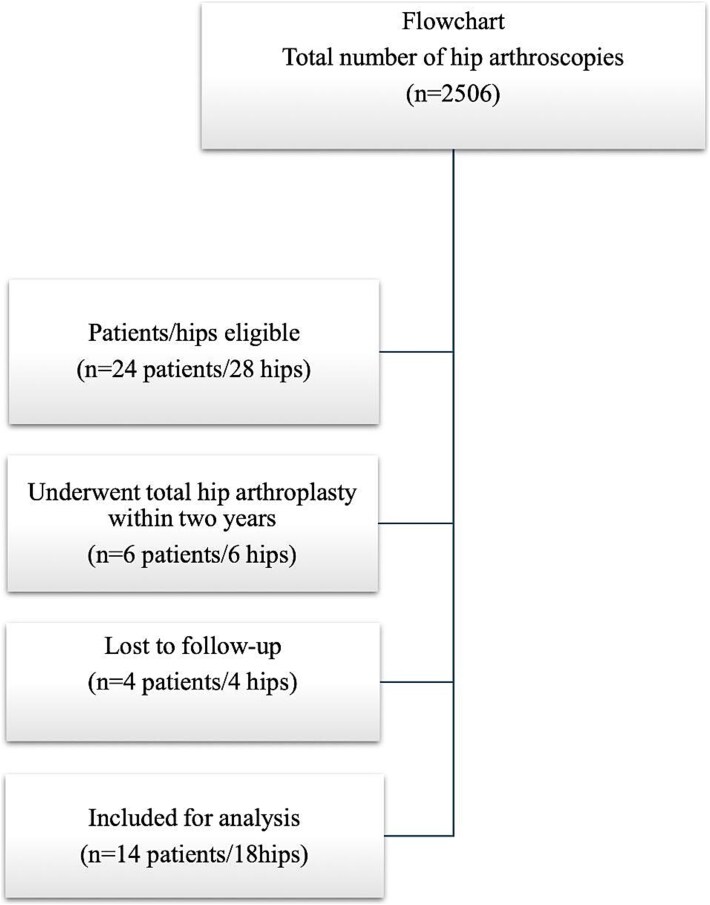
Flowchart of included patients.

In 27 of the 28 hips, a combined cam and pincer resection was performed, and in one patient an isolated pincer resection was performed. In 13 hips the ossified labrum area was resected, and the rest of the labrum was reattached with suture anchors, while in 15 hips labral debridement alone was performed. In this group, 6 out of 24 patients (6 out of 28 hips) underwent a THA within 2 years of the primary hip arthroscopy procedure and four were lost to follow-up and these patients were not included in further analysis (Fig. 1). The mean time to conversion to THA was 11.3 months. Among the six patients that underwent a THA, three had a labral debridement and three had labral damage that was sutured. The average age of this group was 52.6 years (±6 SD) and the average BMI 28.1(±3.2). The radiographic parameters of this group are presented in Table 3. There were no differences in demographics, grade of cartilage lesions, or procedures performed in the group of patients that were lost to follow-up. Among patients included in this study, there were no cases of revision hip arthroscopy or later labral reconstruction.

**Table 3:** Preoperative radiographic parameters of THA group (6 hips).

Alpha angle (degrees)	64 (±2)
CE angle (SD) degrees	33 (±5)
Tönnis angle	12(±5)
Tönnis grade 0 (no hips)	0
Tönnis grade 1 (no hips)	1
Tönnis grade 2 (no hips)	4
Tönnis grade 3 (no hips)	1
Any joint space of 1 (mm)	3
Any joint space of 2 (mm)	2
Any joint space of 3+ (mm)	1

The PROMs scores preoperatively and at 2-year follow-up are presented in Table 4. Comparing PROMs preoperatively with two-year follow-up showed statistically significant improvements (*P* < .05) for: iHOT-12 (39.3 versus 69.1), HAGOS subscales (symptoms 49.0 versus 70.9, pain 53.6 versus 80.0, sport 42.0 versus 68.5, daily activity 53.6 versus 73.2, physical activity 31.7 versus 61.5, quality of life 34.2 versus 65.0), EQ-5D (0.7 versus 0.8) and VAS for overall hip function (44.3 versus 66.5).

**Table 4:** Patient reported outcome scores preoperatively and at minimum 2-year follow-up.

Outcome	Preoperative, mean (SD)	24 months, mean (SD)	Change (SD)	*P*-value	MCID^f^	PASS^g^
iHOT-12^a^	39.3 (15.5)	69.1 (23.4)	29.8 (26.0)	.004	64%	64%
HAGOS^b^—symtoms	49.0 (18.4)	70.9 (21.8)	21.9 (26.7)	.017	57%	64%
HAGOS—pain	53.6 (18.3)	80.0 (17.7)	26.4 (23.9)	.003	64%	71%
HAGOS—daily activity	53.6 (18.9)	73.2 (27.2)	19.6 (29.8)	.035	50%	36%
HAGOS—sports	42.0 (23.0)	68.5 (30.0)	26.6 (31.9)	.012	64%	57%
HAGOS—physical activity	31.7 (29.6)	61.5 (27.2)	29.8 (34.8)	.011	69%	77%
HAGOS—quality of life	34.2 (11.7)	65.0 (23.3)	30.8 (26.8)	.003	69%	85%
EQ-5D^c^	0.7 (0.2)	0.8 (0.1)	0.2 (0.3)	.008	NA	NA
EQ-VAS^d^	63.4 (17.1)	72.5 (15.8)	9.1 (18.6)	.135	NA	NA
HSAS^e^	2.3 (2.0)	3.1 (2.0)	0.8 (2.6)	.304	NA	NA
VAS—overall hip function	44.3 (20.9)	66.5 (24.2)	22.3 (23.4)	.008	NA	NA
Satisfied with surgery (%)		85.7				

a iHOT-12 International Hip Outcome Tool.

b HAGOS Copenhagen Hip and Groin Outcome Score.

c EQ-5D EuroQoL-5 Dimension Questionnaire.

d VAS visual analogue scale.

e Hip Sports & Activity Scale.

f Minimal Clinically Important Difference.

g Patient Acceptable Symptomatic State.

The proportion of patients achieving MCID and PASS for the HAGOS subscales and iHOT-12 were respectively; 57% (MCID) and 64% (PASS) for the symptom subscale, 64% (MCID) and 71% (PASS) for pain, 50% (MCID) and 36% (PASS) for function, 64% (MCID) and 57% (PASS) for sports, 69% (MCID) and 77% (PASS) for physical activity, 69% (MCID) and 85% (PASS) for quality of life, 64% (MCID) and 64% (PASS) for iHOT-12.

At 2-year follow-up, 12 out of 14 patients (85.7%) included in the PROMs analysis reported being satisfied with the surgery.

## DISCUSSION

The most important finding of this study was that patients with FAIS and concomitant LO who underwent hip arthroscopy had an increased risk of conversion to THA at 2 years after surgery. However, patients that did not undergo THA at 2-year follow-up showed a significant improvement of iHOT-12. As LO is a recently described entity, it is important to examine the results of hip arthroscopy in patients with concomitant FAIS and LO. Furthermore, it is interesting to investigate the presence of other comorbidities, such as cartilage lesions or OA. Another aspect to be discussed is the aetiology of LO and how this is correlating or differs to osteophyte formation.

At 2-years follow-up in the current study, 25% of the patients had undergone THA. Most of the patients in this study had high-grade articular cartilage lesions found intraoperatively. This may also explain the high rate of conversion to THA in this group. However, the grade of articular damage was advanced in both the group that had undergone THA at 2-year follow-up and the group that was included in the final PROM analysis (Table 1). Despite the high grade of cartilage lesions, most of the patients that were not operated with THA at the 2-year follow-up achieved improved PROMs values.

The percentage of patients (64%) reaching the iHOT-12 PASS and MCID values in this group shows that clinically meaningful results were achieved. However, variation among the PASS values for the various HAGOS subscales was present. The fact that only 36% achieved the PASS value for the HAGOS-daily activity may depend on the fact that the preoperative value of 53.6 was relatively high compared to the postoperative value of 73.2. Even if the difference was statistically significant, the clinical significance was probably lower. These results support the fact that hip arthroscopy can be beneficial for patients with FAIS and LO, yet more research is needed regarding the indications. Previous studies have shown a higher grade of conversion to THA within 2 years after hip arthroscopy for patients with bipolar cartilage lesions found intraoperatively [30].

To the authors’ knowledge, there exists only one previous study examining the results of hip arthroscopy in patients with FAIS and LO. In a study of Byrd et al. 52 patients with FAIS and LO treated with hip arthroscopy were compared to patients with FAIS without LO [9]. Both groups showed similar improvement in PROMs, however the LO group had more severe symptoms than the control group. Interestingly, there was a lack of signs of joint degeneration, which differs from the patients in the present study. In the study by Byrd et al., mHHS was used as the primary outcome. The use of more modern outcome measures in our study may lead to more valid results [31–34].

**Figure 2:**
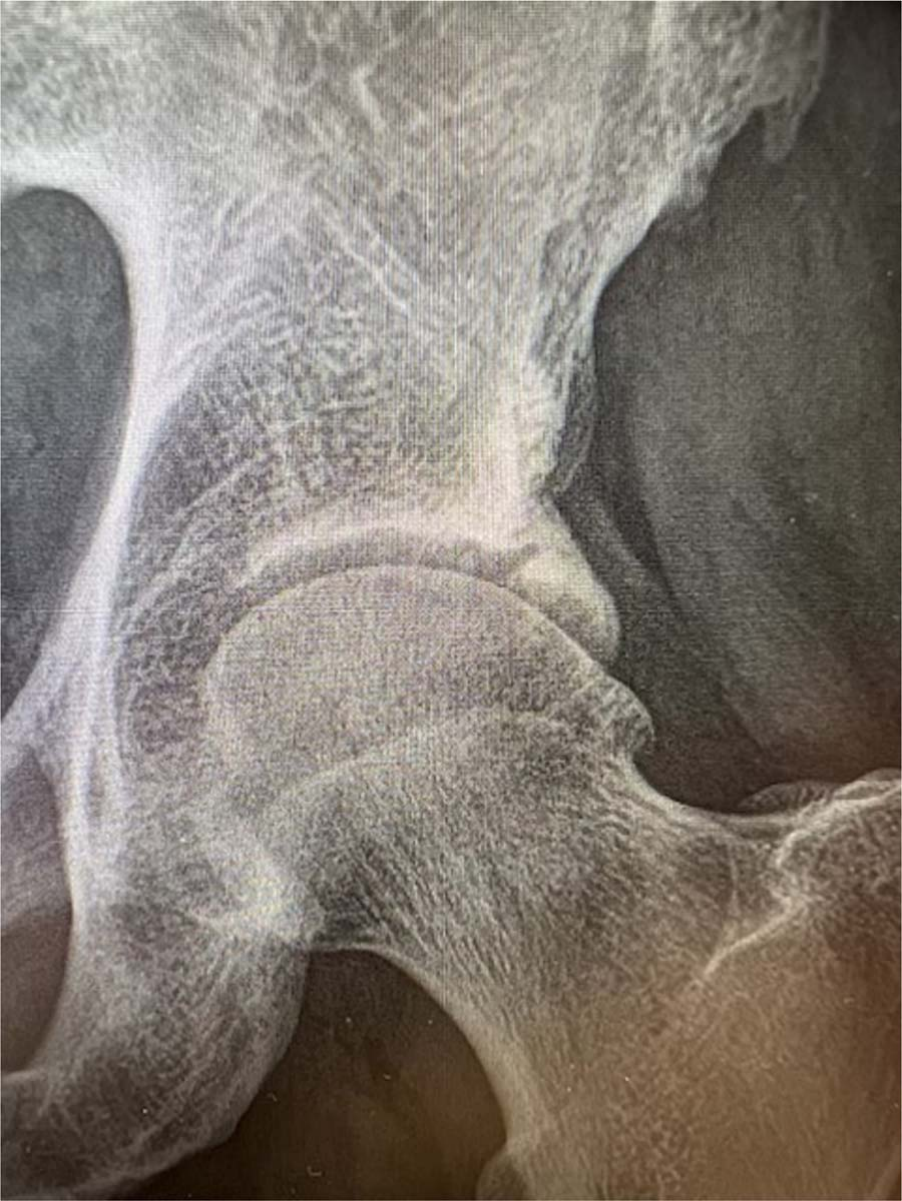
Radiographic image of ossified labrum.

**Figure 3:**
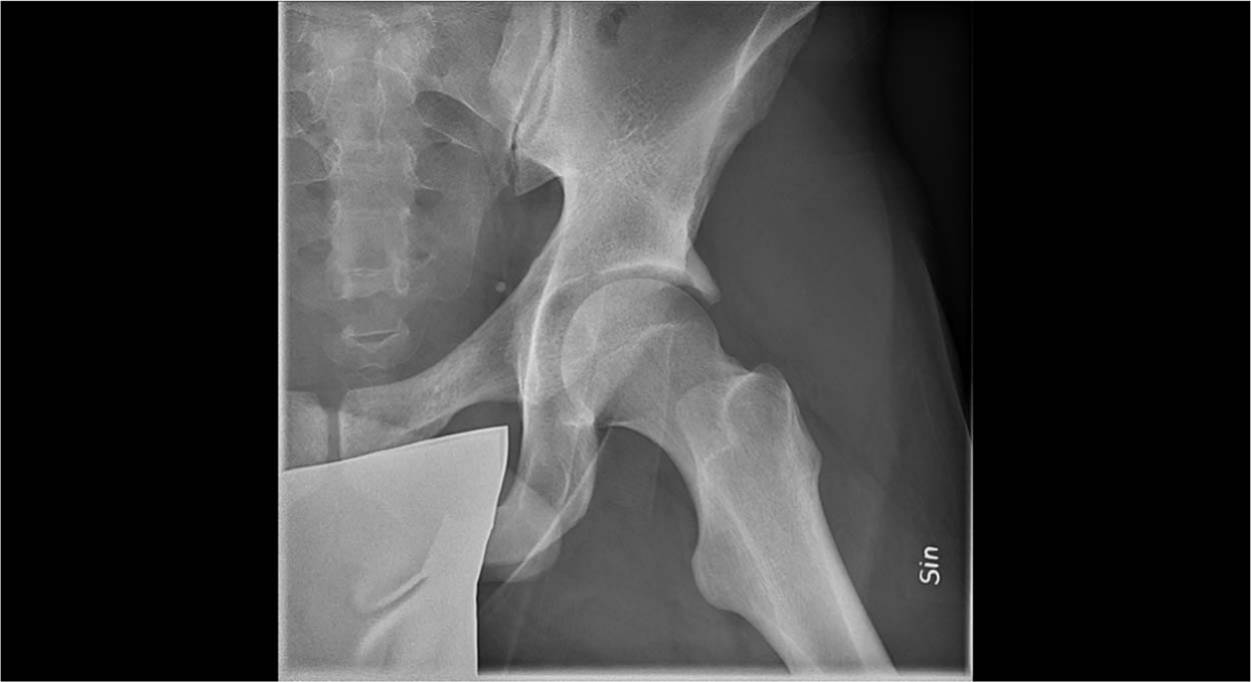
Radiographic image of ossified labrum.

**Figure 4:**
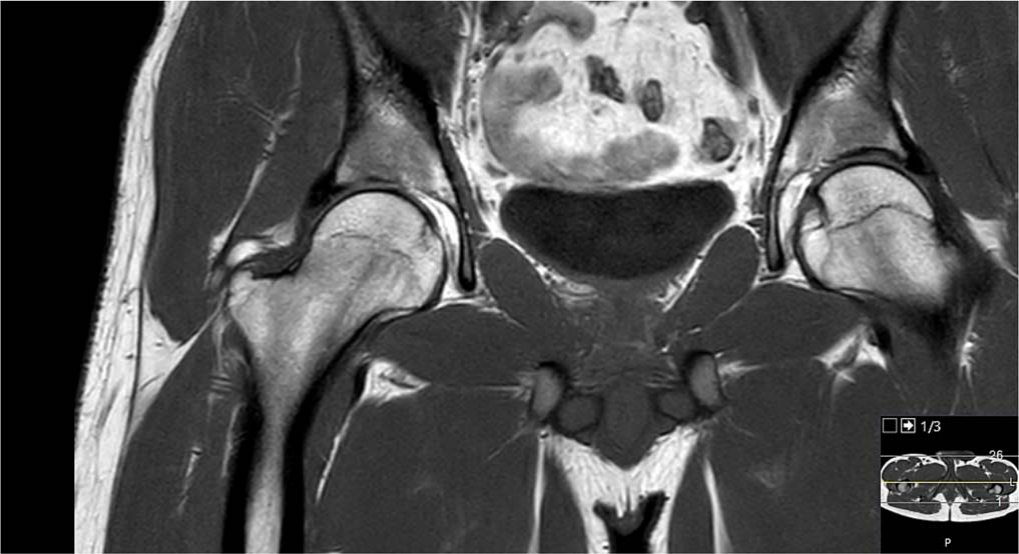
MRI of the same patient with ossified labrum.

**Figure 5:**
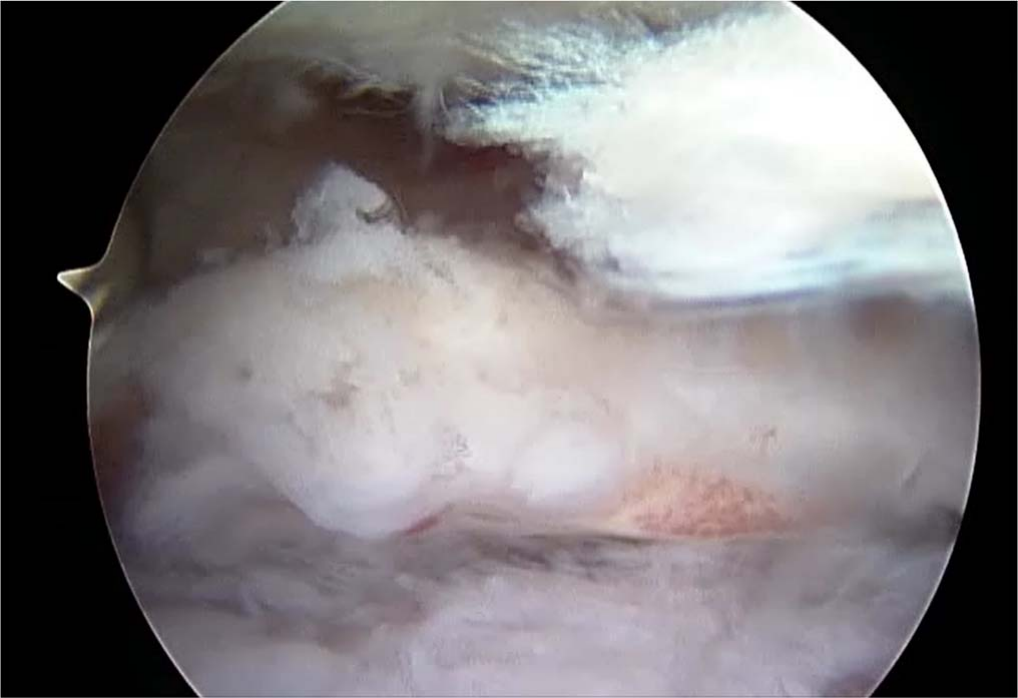
Arthroscopic image of ossified labrum.

**Figure 6:**
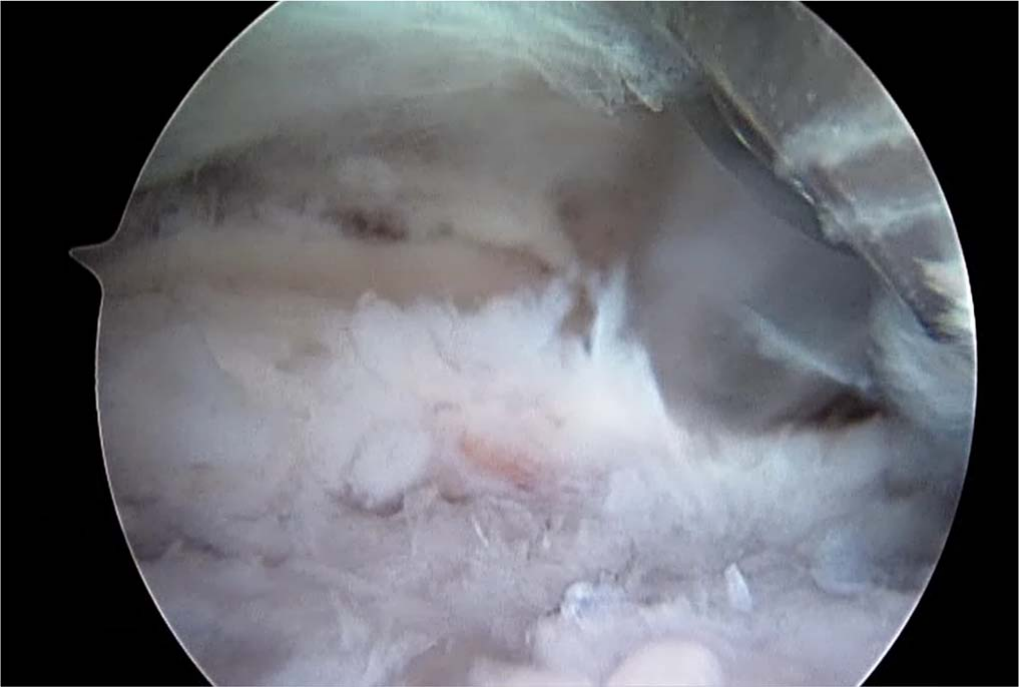
Arthroscopic image of ossified labrum after resection.

The aetiology of LO is still unclear. In a group of patients with coxa profunda, a type of pincer, a histological study of 20 acetabular rims and labrum tissue was performed. It was shown that the labrum in these patients may be displaced and replaced by appositional bone [10]. In later stages of this procedure, the labrum becomes thinner and nearly absent in imaging studies. Even though there were no signs of LO in these samples, the expanding acetabular rim replacing the labrum may represent the same entity. In another histological study of 185 hips with OA, the authors examined the osteophyte formed at the area of the craniolateral acetabular rim [14]. Signs of metaplasia of the labral tissue or synovial membrane, as well as a form of chondral ossification, were found in these tissue samples. In the patient group with FAIS and LO examined in the study of Byrd et al. the histological analysis of the LO areas showed both a bony extension of the acetabular rim and areas of enchondral ossification of the labrum [9]. More studies in this field are needed to clarify the pathogenesis of LO and its relation to osteophyte formation.

There are several limitations to this study. One is the short follow-up period of 2 years; long-term results on this specific patient group are warranted. Another limitation is the small group of 24 patients as well as the fact that 4 patients were lost to follow-up and 6 patients had undergone THA at 2-years after surgery. Moreover, the patients included in this study were selected out of a geographically limited registry. This fact can potentially affect the generalizability of the conclusions drawn from this study, as genetical factors and local healthcare system factors could have an impact on the population studied. Another limitation is that the amount of ossified labrum was not recorded in our registry, since there is no validated quantifying scale. This is a retrospective analysis of prospectively collected data; hence, a power analysis was not conducted prior to this study, increasing the risk for a type II error. However, this is to our knowledge, one of the few studies evaluating the surgical results for FAIS and concomitant LO with modern PROMs.

## CONCLUSION

In this patient group, 6 out of 24 patients underwent THA within 2 years from the primary hip arthroscopy. Patients with FAIS and ossified labrum treated with hip arthroscopy that had not undergone THA at 2-year follow-up had improved outcomes in hip related PROMs.

## Data Availability

The data underlying this article will be shared upon reasonable request to the corresponding author.

## References

[1] Grantham WJ, Philippon MJ. Etiology and Pathomechanics of Femoroacetabular impingement. Curr Rev Musculoskelet Med 2019;12:253–9. 10.1007/s12178-019-09559-1 31278564 PMC6684670

[2] Hadeed MM, Cancienne JM, Gwathmey FW. Pincer impingement. Clin Sports Med 2016;35:405–18. 10.1016/j.csm.2016.02.003 27343393

[3] Ganz R, Parvizi J, Beck M et al. Femoroacetabular impingement: a cause for osteoarthritis of the hip. Clin Orthop Relat Res 2003;417:112–20. 10.1097/01.blo.0000096804.78689.c2 14646708

[4] Kopec JA, Cibere J, Li LC et al. Relationship between physical activity and hip pain in persons with and without cam or pincer morphology: a population-based case-control study. Osteoarthr Cartil 2017;25:1055–61. 10.1016/j.joca.2017.02.795 28219714

[5] Raveendran R, Stiller JL, Alvarez C et al. Population-based prevalence of multiple radiographically-defined hip morphologies: the Johnston County osteoarthritis project. Osteoarthr Cartil 2018;26:54–61. 10.1016/j.joca.2017.10.002 PMC5732866 29024801

[6] Bsat S, Frei H, Beaulé PE. The acetabular labrum: a review of its function. Bone Joint J 2016;98-B:730–5. 10.1302/0301-620X.98B6.37099 27235512

[7] Faber BG, Ebsim R, Saunders FR et al. Osteophyte size and location on hip DXA scans are associated with hip pain: findings from a cross sectional study in UK biobank. Bone 2021;153:116146. 10.1016/j.bone.2021.116146 34389476 PMC8503366

[8] Azuma H, Kako K, Itoigawa Y. Aging process of the acetabulum with special reference to the osteophyte formation (author's transl). Nihon Seikeigeka Gakkai Zasshi 1980;54:153–60. 7391621

[9] Byrd JW, Jones KS, Freeman CR. Surgical outcome of pincer femoroacetabular impingement with and without labral ossification. Arthroscopy 2016;32:1022–9. 10.1016/j.arthro.2015.12.042 26968308

[10] Corten K, Ganz R, Chosa E et al. Bone apposition of the acetabular rim in deep hips: a distinct finding of global pincer impingement. J Bone Joint Surg Am 2011;93 Suppl 2:10–6. 10.2106/JBJS.J.01799 21543682

[11] Dumont GD, Menge TJ, Money AJ et al. Para-acetabular radiopaque densities in patients with femoroacetabular impingement: a retrospective assessment of prevalence and characteristics. Orthop J Sports Med 2020;8:2325967119892330. 10.1177/2325967119892330 32030344 PMC6978822

[12] Ninomiya S, Shimabukuro A, Tanabe T et al. Ossification of the acetabular labrum. J Orthop Sci 2000;5:511–4. 10.1007/s007760070031 11180910

[13] Dudaric L, Dumic-Cule I, Divjak E et al. Bone remodeling in osteoarthritis-biological and radiological aspects. Medicina (Kaunas) 2023;59:1613. 10.3390/medicina59091613 37763732 PMC10537088

[14] Hotta Y, Matsui K, Nakada D et al. The natural course of osteoarthritis of the hip. Indication of conservative treatment in relation to osteophyte formation at the acetabular rim. Nihon Seikeigeka Gakkai Zasshi 1985;59:1–15. 4009006

[15] Valente C, Haefliger L, Favre J et al. Ossification of the acetabular rim: a highly prevalent finding in asymptomatic non-osteoarthritic hips of all ages. Eur Radiol 2021;31:6802–9. 10.1007/s00330-021-07750-y 33715089 PMC8379116

[16] Griffin DR, Parsons N, Mohtadi NG et al. A short version of the International Hip Outcome Tool (iHOT-12) for use in routine clinical practice. Arthroscopy 2012;28:611–6 quiz 616-618. 10.1016/j.arthro.2012.02.027 22542434

[17] Rabin R, de Charro F. EQ-5D: a measure of health status from the EuroQol group. Ann Med 2001;33:337–43. 10.3109/07853890109002087 11491192

[18] Thomeé R, Jónasson P, Thorborg K et al. Cross-cultural adaptation to Swedish and validation of the Copenhagen Hip and Groin Outcome Score (HAGOS) for pain, symptoms and physical function in patients with hip and groin disability due to femoro-acetabular impingement. Knee Surg Sports Traumatol Arthrosc 2014;22:835–42. 10.1007/s00167-013-2721-7 24146052

[19] Thorborg K, Hölmich P, Christensen R et al. The Copenhagen Hip and Groin Outcome Score (HAGOS): development and validation according to the COSMIN checklist. Br J Sports Med 2011;45:478–91. 10.1136/bjsm.2010.080937 21478502

[20] Konan S, Rayan F, Meermans G et al. Validation of the classification system for acetabular chondral lesions identified at arthroscopy in patients with femoroacetabular impingement. J Bone Joint Surg Br 2011;93-B:332–6. 10.1302/0301-620X.93B3.25322 21357954

[21] Martin HD, Savage A, Braly BA et al. The function of the hip capsular ligaments: a quantitative report. Arthroscopy 2008;24:188–95. 10.1016/j.arthro.2007.08.024 18237703

[22] Sansone M, Ahldén M, Jónasson P et al. Outcome after hip arthroscopy for femoroacetabular impingement in 289 patients with minimum 2-year follow-up. Scand J Med Sci Sports 2017;27:230–5. 10.1111/sms.12641 26791778

[23] Thaunat M, Murphy CG, Chatellard R et al. Capsulotomy first: a novel concept for hip arthroscopy. Arthrosc Tech 2014;3:e599–603. 10.1016/j.eats.2014.06.016 25473614 PMC4246391

[24] Matsuda DK . Acute iatrogenic dislocation following hip impingement arthroscopic surgery. Arthroscopy 2009;25:400–4. 10.1016/j.arthro.2008.12.011 19341927

[25] Sansone M, Ahldén M, Jónasson P et al. Total dislocation of the hip joint after arthroscopy and ileopsoas tenotomy. Knee Surg Sports Traumatol Arthrosc 2013;21:420–3. 10.1007/s00167-012-2300-3 23179452

[26] Nwachukwu BU, Beck EC, Kunze KN et al. Defining the clinically meaningful outcomes for arthroscopic treatment of femoroacetabular impingement syndrome at minimum 5-year follow-up. Am J Sports Med 2020;48:901–7. 10.1177/0363546520902736 32167843

[27] Ishøi L, Thorborg K, Ørum MG et al. How many patients achieve an acceptable symptom state after hip arthroscopy for femoroacetabular impingement syndrome? A cross-sectional study including PASS cutoff values for the HAGOS and iHOT-33. Orthop J Sports Med 2021;9:2325967121995267. 10.1177/2325967121995267 33889644 PMC8040572

[28] Jónasson P, Baranto A, Karlsson J et al. A standardised outcome measure of pain, symptoms and physical function in patients with hip and groin disability due to femoro-acetabular impingement: cross-cultural adaptation and validation of the international Hip Outcome Tool (iHOT12) in Swedish. Knee Surg Sports Traumatol Arthrosc 2014;22:826–34. 10.1007/s00167-013-2710-x 24136045

[29] Nwachukwu BU, Chang B, Beck EC et al. How should we define clinically significant outcome improvement on the iHOT-12? HSS J 2019;15:103–8. 10.1007/s11420-018-9646-0 31327939 PMC6609659

[30] Ruzbarsky JJ, Seiter MN, Soares R et al. Lower center edge angle and Bioipolar cartilage lesions are associated with conversion to hip arthroplasty within 2 years following hip arthroscopy: a matched cohort analysis. Arthroscopy 2022;38:1480–5. 10.1016/j.arthro.2021.09.025 34601009

[31] Gasparin GB, Frasson VB, Fritsch CG et al. Are the Harris hip score and the hip outcome score valid patient-reported outcome measures for femoroacetabular impingement syndrome? Braz J Phys Ther 2022;26:100422. 10.1016/j.bjpt.2022.100422 35696813 PMC9193913

[32] Griffin DR, Dickenson EJ, O'Donnell J et al. The Warwick agreement on femoroacetabular impingement syndrome (FAI syndrome): an international consensus statement. Br J Sports Med 2016;50:1169–76. 10.1136/bjsports-2016-096743 27629403

[33] Kivlan BR, Martin RL, Christoforetti JJ et al. The patient acceptable symptomatic state of the 12-item International Hip Outcome Tool at 1-year follow-up of hip-preservation surgery. Arthroscopy 2019;35:1457–62. 10.1016/j.arthro.2018.11.072 31000393

[34] Ueland TE, Disantis A, Carreira DS et al. Patient-reported outcome measures and clinically important outcome values in hip arthroscopy: a systematic review. JBJS Rev 2021;9:e20.00084. 10.2106/JBJS.RVW.20.00084 33512970

